# Supplementary material for: A minimal gene set characterizes TIL specific for diverse tumor antigens across different cancer types
Source: Nat Commun. 2025 Feb 3;16:1070. doi: 10.1038/s41467-024-55059-3 (PMC11791090; doi:10.1038/s41467-024-55059-3)
Supplement: Supplementary file 1 — Supplementary Information [file 41467_2024_55059_MOESM1_ESM.pdf]

A minimal gene set characterizes TIL specific for diverse tumor antigens across different cancer types

Zhen Zeng[1,2,3], Tianbei Zhang[1,2,3], Jiajia Zhang[4], Shuai Li[5], Sydney Connor[1,2,3], Boyang Zhang[5], Yimin Zhao[5,ϕ], Jordan Wilson[5], Dipika Singh[1,2,3], Rima Kulikauskas[6,7], Candice D. Church[6,7], Thomas H. Pulliam[6,7], Saumya Jani[6,7], Paul Nghiem[6,7], Suzanne L. Topalian[1,3,8], Patrick M. Forde[1,3], Drew M. Pardoll[1,2,3], Hongkai Ji[5,#], Kellie N. Smith[1,2,3,#,*]

Affiliations

[1]Bloomberg~Kimmel Institute for Cancer Immunotherapy, Baltimore, MD

[2]Mark Center for Advanced Genomics and Imaging, Baltimore, MD

[3]Sidney Kimmel Comprehensive Cancer Center, Baltimore, MD

[4]David Geffen School of Medicine, University of California, Los Angeles, CA

[5]Department of Biostatistics, Johns Hopkins Bloomberg School of Public Health, Baltimore, MD

[6]Fred Hutchinson Cancer Center, Seattle, WA

[7]Department of Medicine, University of Washington, Seattle, WA

[8]Department of Surgery, Johns Hopkins University School of Medicine

[ϕ]Present address: Department of Biostatistics, University of Washington, Seattle, WA.

[#]Equal contribution

*Corresponding author:

CRB1, room 4M51

1650 Orleans Street

Baltimore, MD 21287

kellie@jhmi.edu, 410-502-7523

Supplementary Figures

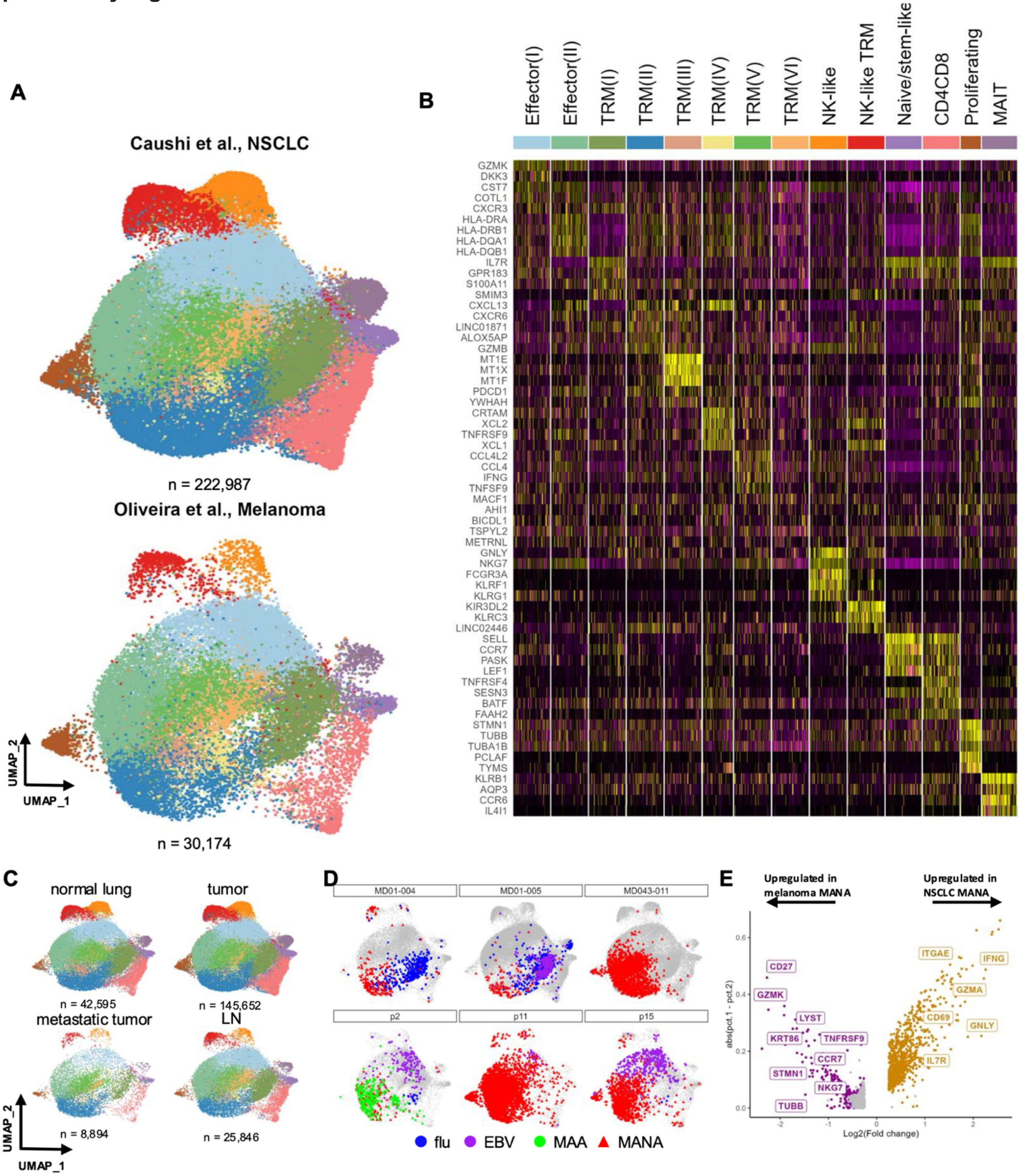

Supplementary Figure 1. Integration of published NSCLC and melanoma datasets. (**A**) Relative expression of top 5 most differentially expressed genes, 5,000 cells (or all cells in the cluster if there are less than 5,000 cells in the cluster) were randomly selected from each cluster for visualization. (**B**) UMAP projection split by cancer types, colored by different clusters. (**C**) UMAP projection split by NSCLC tissue types, colored by different clusters. There are four tissue types for NSCLC (normal, tumor, tumor form metastasis sample and LN). (**D**) Overlay of antigen-specific T cells on UMAP of six patients (with more than 30 MANA-specific T cell identified), including 3 from NSCLC and 3 from melanoma. MANA-, MAA-, EBV- and InfluenzaA-specific cells are colored by red, green, blue, purple.

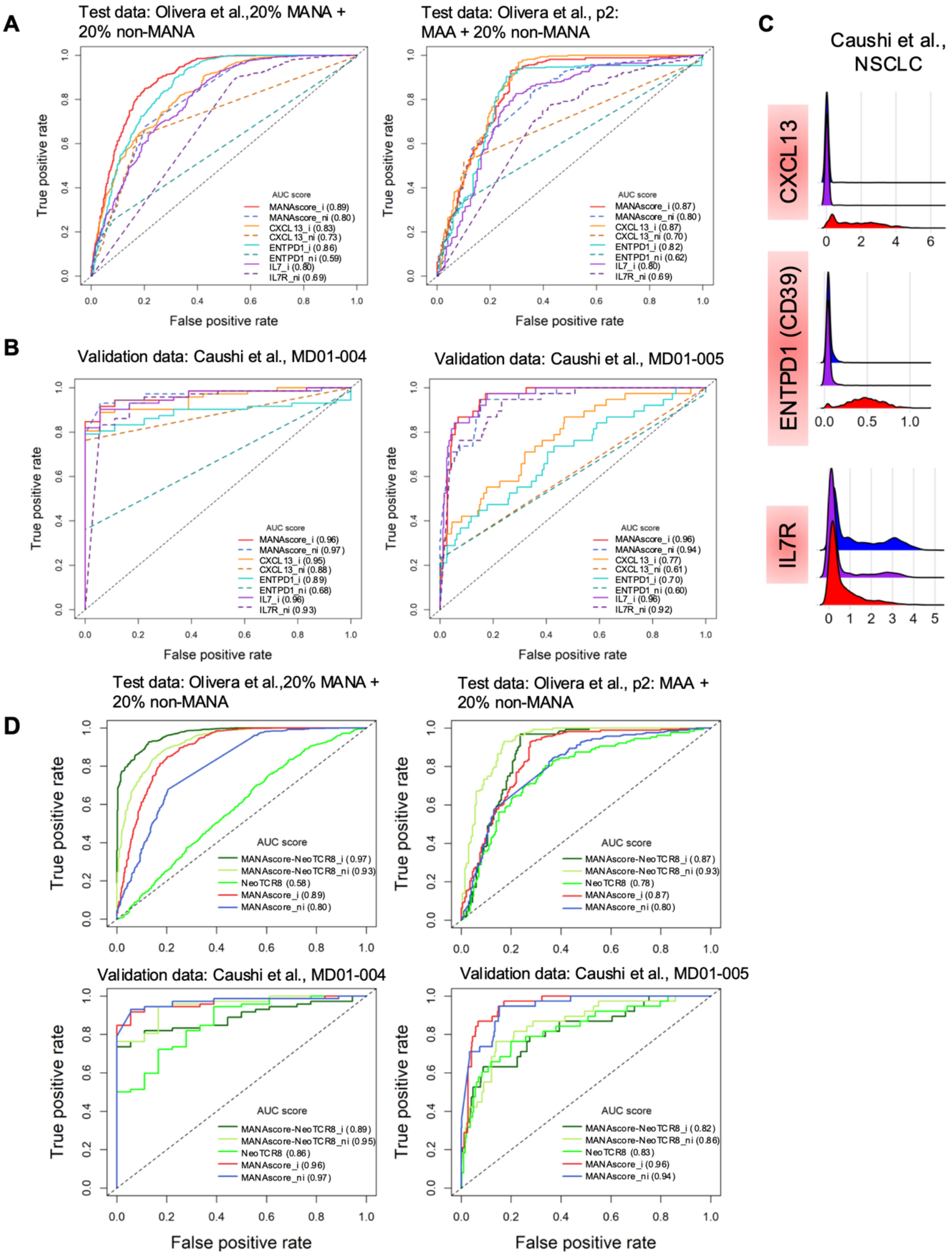

Supplementary Figure 2. Development of the score from published NSCLC and melanoma data. (A) Receiver operating characteristic (ROC) curves for the performance of imputation/non-imputation combine voting models, single gene models and two gene models on melanoma test data (20% MANA- and EBV-/InfluenzaA-specific T cells), and melanoma associated antigen- (MAA-) and 20% EBV-/InfluenzaA- (test data) specific T cells in one melanoma patient p2. **(B)** ROC curves for the performance of imputation/non-imputation combine voting models and single gene models and two gene models on validation dataset, MD01-004 and MD01-005 from Caushi et al., NSCLC cohort. (**C**) Expression level of three genes previously reported to mark tumor-reactive vs bystander CD8+ TIL in validated MANA-, EBV- and InfluenzaA-specific TIL in the Caushi et al NSCLC dataset. **(D)** ROC curves for the performance of imputation/non-imputation combined voting MANAscore models, NeoTCR8 models (MANAscore-NeoTCR8), and original scGSEA NeoTCR8 models on melanoma test data and lung cancer validation data.

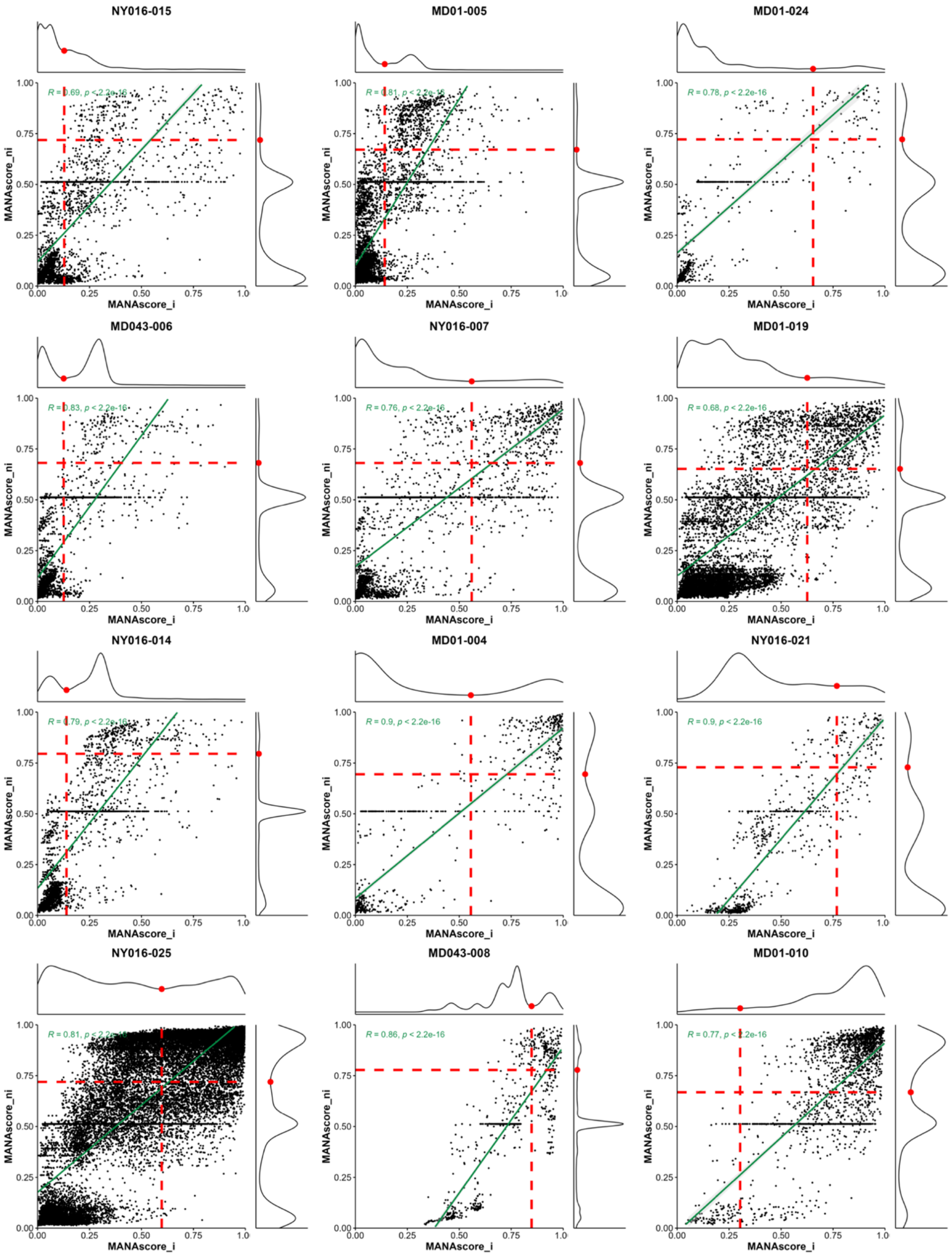

Supplementary Figure 3. Scatter plot of imputation MANAscore and non-imputation MANAscore in NSCLC patient tumors, cutoffs were set for define MANAscore^{hi} T cells by distribution of these two scores, the linear correlation of these two scores was marked in green, Pearson's correlation coefficient is added.

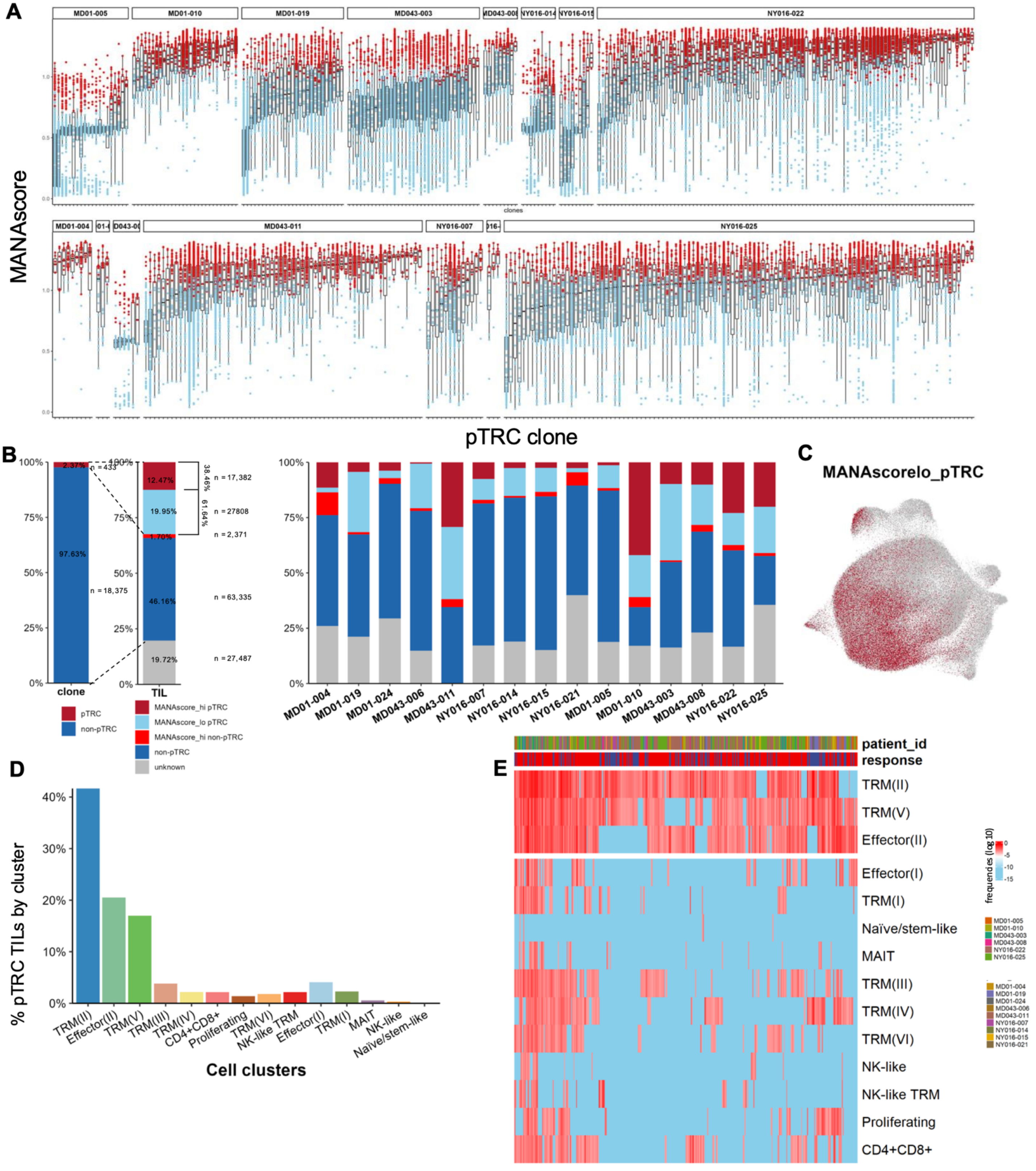

Supplementary Figure 4. Characteristics of pTRC. (A) MANAscore distribution of all pTRC clones (n= 443), MANAscore^{hi} TIL were colored by red, and MANAscore^{lo} TIL were colored by blue, the pTRCs in each patient were ranked by the median MANAscore within the clone. **(B)** Category contribution for clone and TIL. **(C)** MANAscore^{lo} TIL pTRCs were overlaid on the CD8+ UMAP in Fig. 1B. **(D)** Cell type contribution to all pTRC identified. **(E)** Clonal sharing pattern of pTRC.

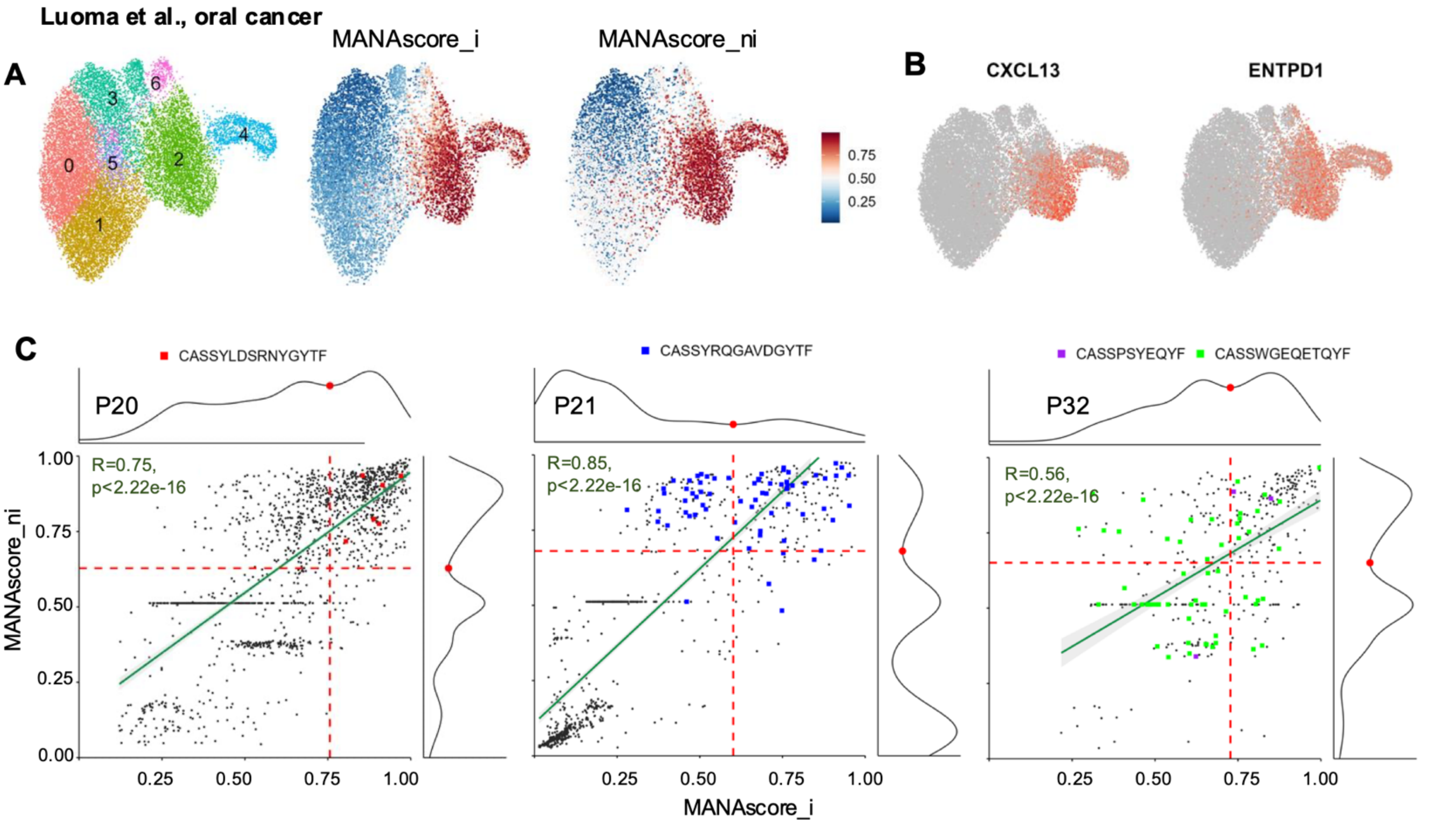

Supplementary Figure 5. Application of MANAscore on independent validation dataset of Luoma et al. oral cancer cohort. (A) MANAscore predicted for CD8+ TIL in Luoma et al. oral cancer cohort, (**B**) Expression of CXCL13 and ENTPD1 on the UMAP, (C) Scatter plot of MANAscore_i and MANAscore_ni for CD8+ TIL in 3 patients from the Luoma et al. oral cancer cohort, 4 functionally validated T cell clones identified were highlighted. Cutoffs were set to define MANAscore^{hi} CD8+ TIL by distribution of these two scores, the linear correlation of these two scores was marked in green, Pearson's correlation coefficient is added.

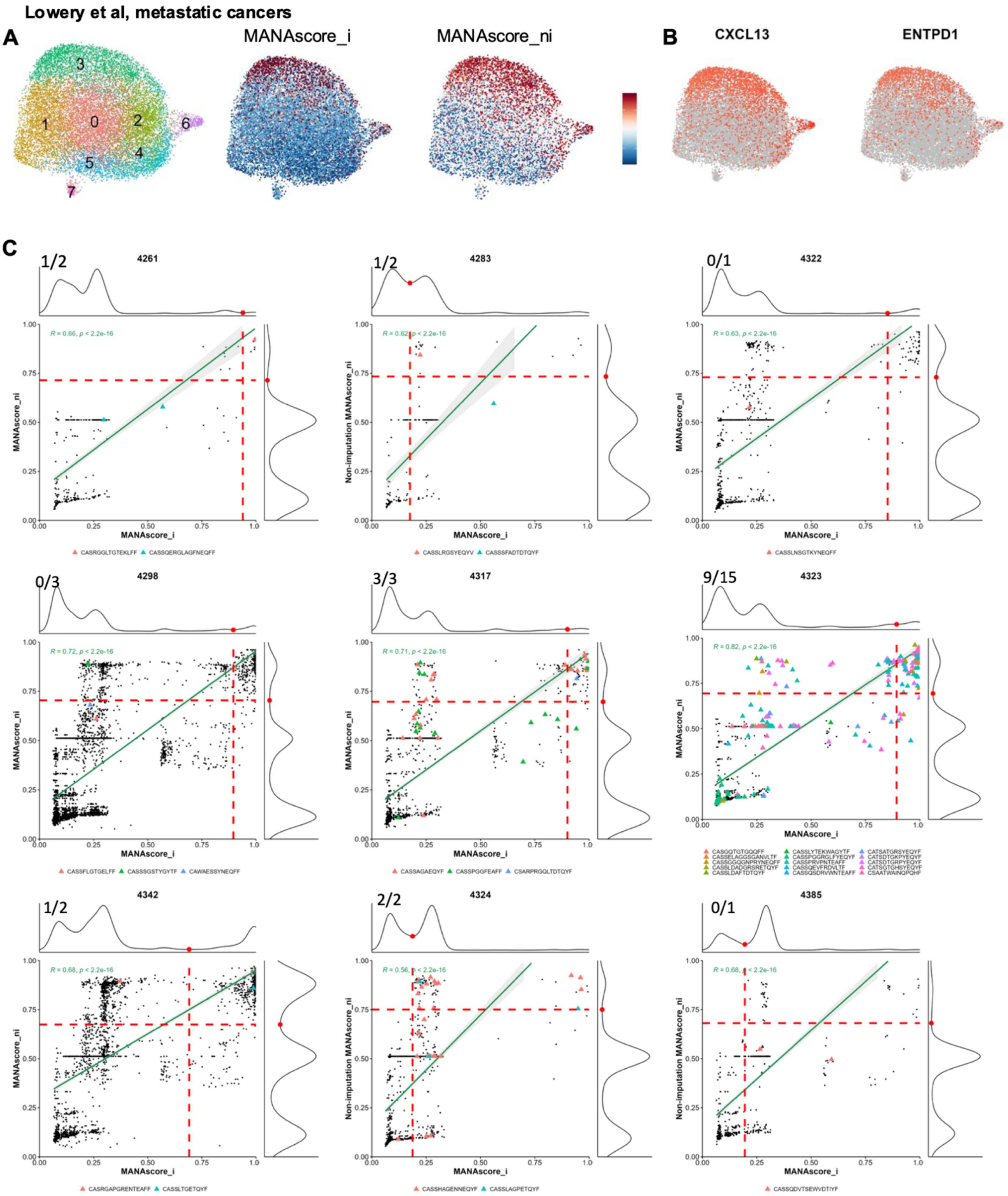

Supplementary Figure 6. Application of MANAscore on independent validation dataset of Lowery et al. metastatic cancer cohort. (A) MANAscore predicted for CD8+ TIL in Lowery et al. metastatic cancer cohort. (B) Expression of CXCL13 and ENTPD1 on the UMAP, **(C)** Scatter plot of MANAscore_i and MANAscore_ni in metastatic patient tumors, cutoffs were set for define MANAscore^{hi} T cells by distribution of these two scores, the linear correlation of these two scores was marked in green, Pearson's correlation coefficient is added. The known neoantigen-specific TIL were colored by the clone type.

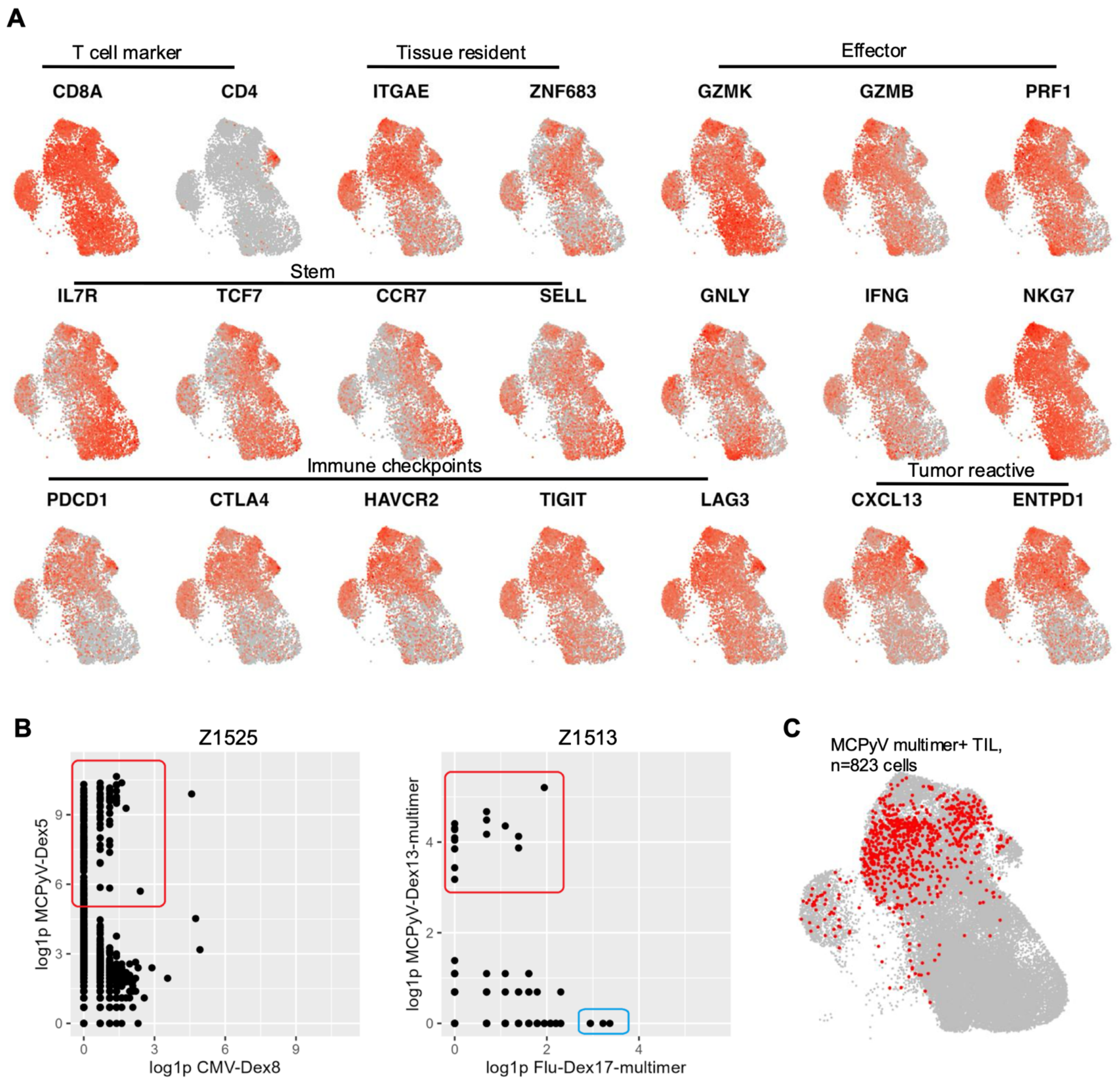

Supplementary Figure 7. MCPyV-specific T cell in Merkel cell carcinoma identified by CITE-seq. (A) Expression of selected genes. (**B**) Scatter plot of multimer read count in three MCPyV$^+$ Merkel cell carcinoma patients. **(C)** CITEseq identified MCPyV-specific multimer+ TIL (n=823 cells) overlaid on the CD8+ TIL UMAP of Merkel cell carcinoma cohort from patients with MCPyV positive.

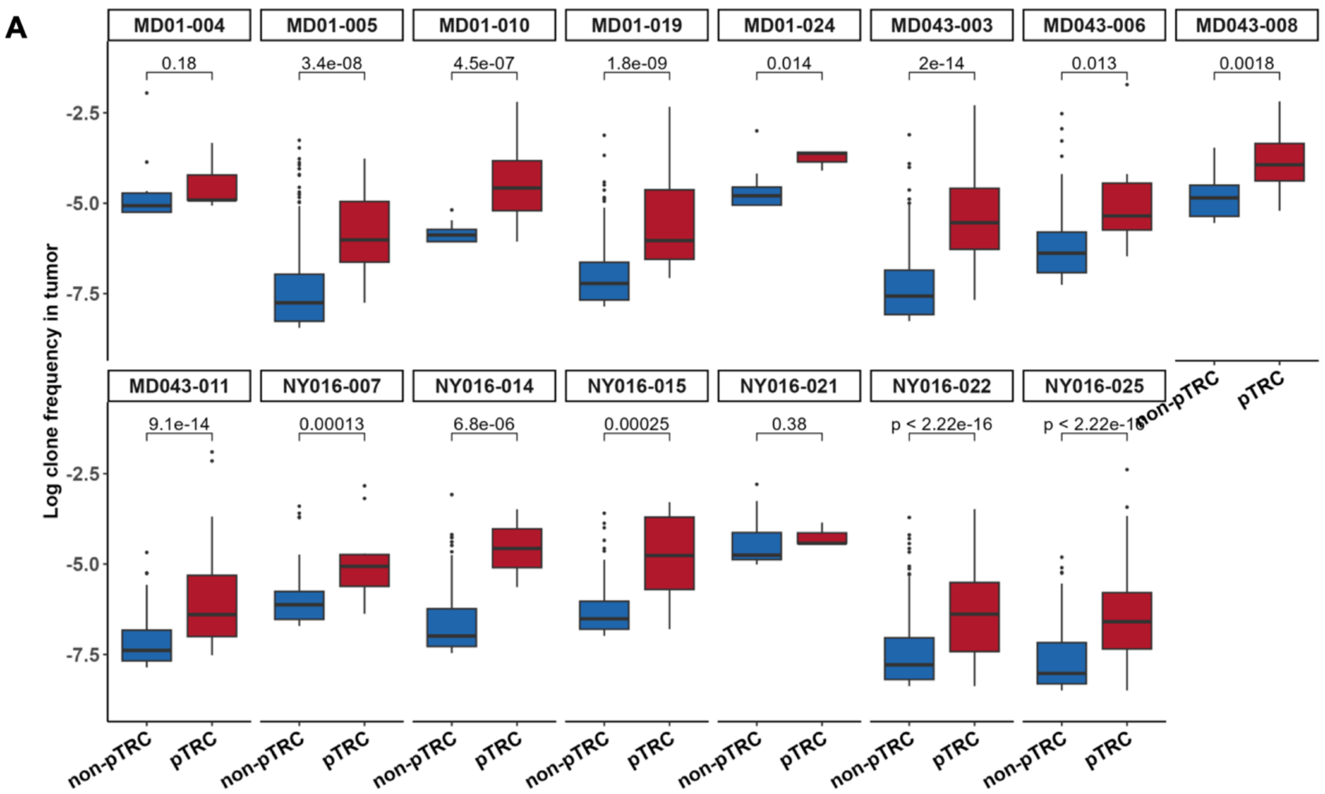

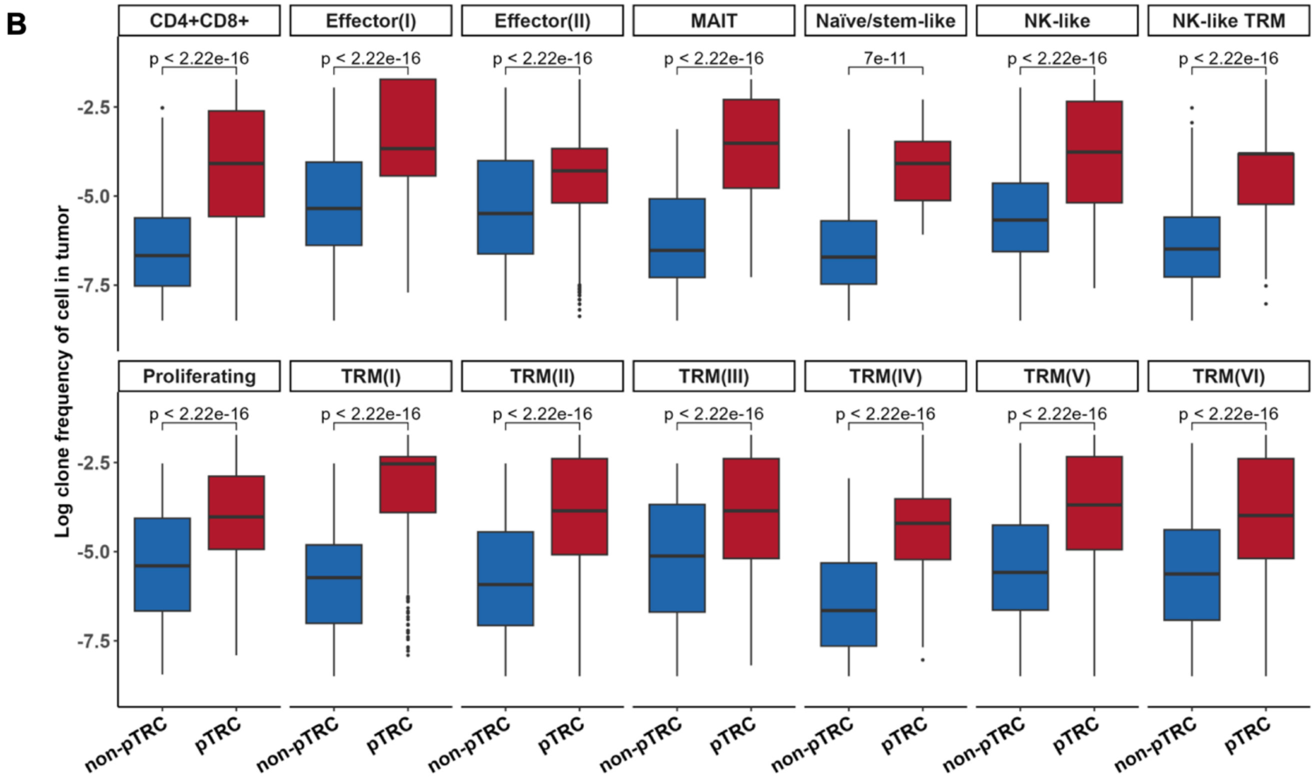

Supplementary Figure 8. Comparison between pTRC and non-pTRC. (A) Boxplot of frequencies of pTRC and non-pTRC in each patient tumor. **(B)** Boxplot of frequencies of pTRC and non-pTRC in each cluster. The comparisons were performed using two-sided Wilcoxon rank-sum test. Box plots represent minima maxima as whiskers, the median as the center line within each box, and the interquartile range (IQR) between 25th and 75th percentiles as the bounds of each box.

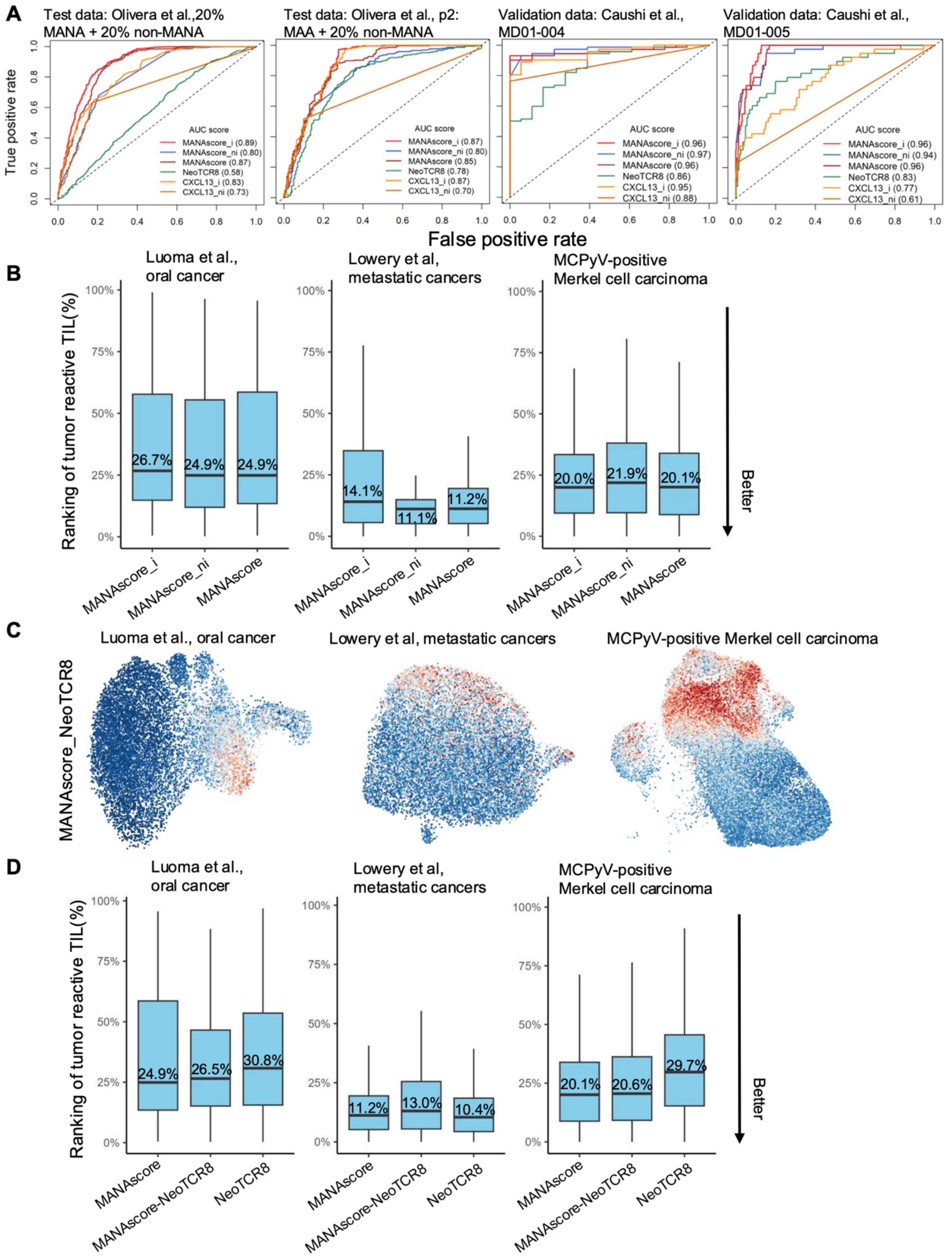

Supplementary Figure 9. Performance of different models. (A) ROC curves for the performance of imputation/non-imputation combined voting MANAscore models, overall MANAscore, original scGSEA NeoTCR8 models and imputation/non-imputation CXCL13 single gene models on melanoma test data and lung cancer validation data. **(B)** Rankings of MANAscore_i, MANAscore_ni and overall MANAscore of tumor reactive TIL in three datasets, including Luoma et al. oral cancer, Lowery et al. metastatic cancers, and our novel MCPyV-positive Merkel cell carcinoma dataset. **(C)** Combined voting NeoTCR8 score for TIL in three datasets. **(D)** Rankings of MANAscore, MANAscore-NeoTCR8 (using combined voting model), and the original NeoTCR8 gsea score of tumor reactive TIL in these three datasets. The median ranking is shown; smaller ranking represents higher score.

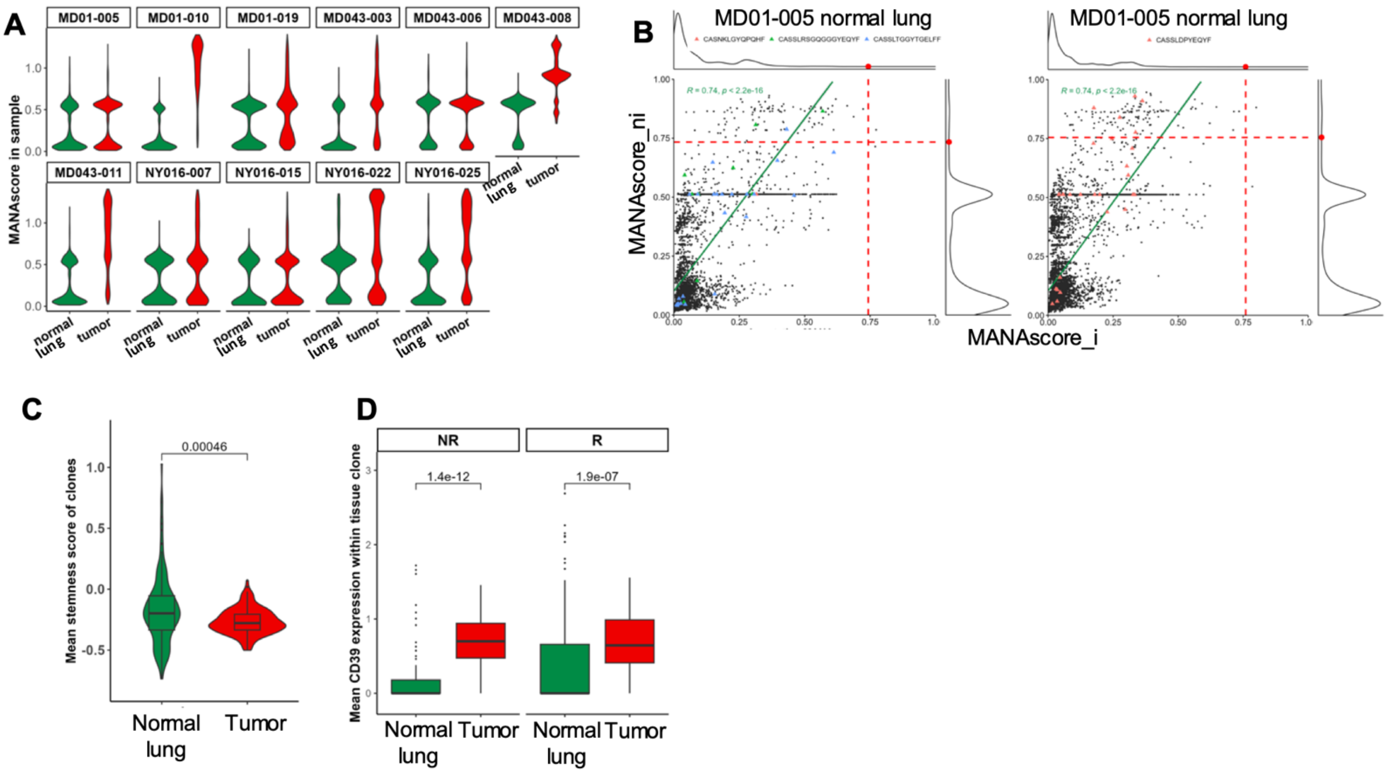

Supplementary Figure 10. Comparison of pTRCs/non-pTRCs in tissue compartments. (A) Violin plot of MANAscore ($\sqrt{\text{MANAscore\_i}^2 + \text{MANAscore\_ni}^2}$) in tumor and normal for different patients. **(B)** Scatter plot of imputation MANAscore and non-imputation MANAscore in MD01-005 and MD043-011 normal tissues, cutoffs were set for defining MANAscore^{hi} T cells by distribution of these two scores, the linear correlation of these two scores was marked in green, Pearson's correlation coefficient is added. The validated MANA-specific T cells were highlighted. **(C)** Average stemness scores in pTRCs in normal and tumor tissues. **(D)** Mean expression of CD39 of shared pTRC in normal and tumor tissue, divided by response status. The comparisons in C-D were performed using two-sided Wilcoxon rank-sum test.

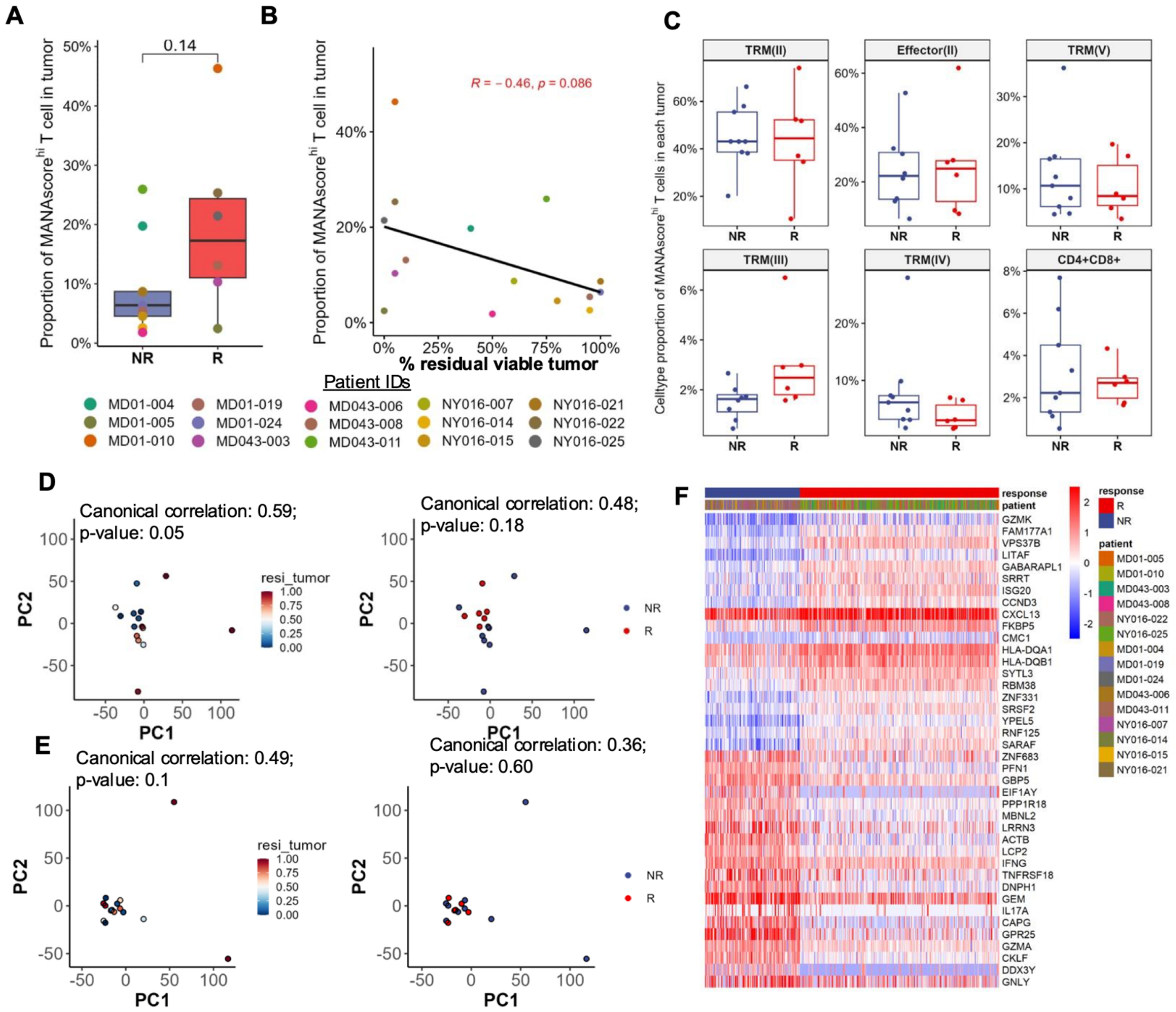

Supplementary Figure 11. Comparison of MANAscore^{hi} pTRCs or pTRCs in responder and non-responders. (A) Proportion of MANAscore^{hi} pTRC between responder and non-responder. The comparison was performed using two-sided Wilcoxon rank-sum test. **(B)** Correlation between the proportion of MANAscore^{hi} pTRC TIL and residual viable tumor of each patient. **(C)** Cell type proportion pTRC TIL in responder and non-responder patients. **(D)** PCA of pseudobulk gene expression for MANAscore^{hi} pTRC TIL in individual patients with percentage of residual viable tumors (left) and with different response status (right). **(E)** PCA of pseudobulk gene expression for pTRC TIL in individual patients with percentage of residual viable tumors (left) and with different response status (right). **(F)** Top 20 differentially expressed genes in pTRC of responder and non-responder patients based on pseudobulk clone differentially gene expression analysis.

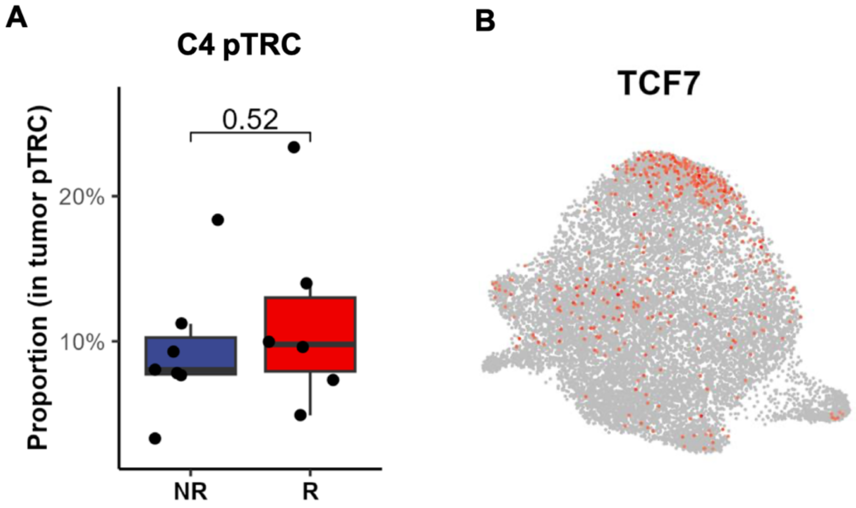

Supplementary Figure 12. Subclustering of pTRC. (A) Boxplot of proportion of C1 and C5 pTRC in all responding and non-responding pTRC. The comparison was performed using two-sided Wilcoxon rank-sum test. **(B)** Feature plot of TCF7 on the UMAP of pTRC.
