# Supplementary material for: A minimal gene set characterizes TIL specific for diverse tumor antigens across different cancer types
Source: Nat Commun. 2025 Feb 3;16:1070. doi: 10.1038/s41467-024-55059-3 (PMC11791090; doi:10.1038/s41467-024-55059-3)
Supplement: Supplementary file 2 — Description of Additional Supplementary Files [file 41467_2024_55059_MOESM2_ESM.pdf]

Description of Additional Supplementary Files

**Supplementary Data 1**	Patient clinal information
**Supplementary Data 2**	Validatated MANA-specific TIL in resectable NSCLC and melanoma
**Supplementary Data 3**	Cluster markers for CD8+ TIL in integrated melanoma and NSCLC datasets
**Supplementary Data 4**	Cluster cell number in NSCLC and melanoma tissues
**Supplementary Data 5**	Numbers and proportions of NSCLC and melanoma MANA-specific TIL in each cluster
**Supplementary Data 6**	MANAscorehi TIL in each NSCLC patient
**Supplementary Data 7**	pTRC/non-pTRC identified in NSCLC tumor
**Supplementary Data 8**	Multimers tested in virus-positive MCC
**Supplementary Data 9**	Cluster markers for CD8+ TIL in Merkel cell carcinoma cohort
**Supplementary Data 10**	pTRC/non-pTRC identified in Merkel cell carcinoma cohort
**Supplementary Data 11**	Differentially expressed genes in pTRC and non-pTRC
**Supplementary Data 12**	Gene signatures
**Supplementary Data 13**	Differentially expressed genes of pTRC in responders and non-responders
