# Supplementary material for: A minimal gene set characterizes TIL specific for diverse tumor antigens across different cancer types
Source: Nat Commun. 2025 Feb 3;16:1070. doi: 10.1038/s41467-024-55059-3 (PMC11791090; doi:10.1038/s41467-024-55059-3)
Supplement: Supplementary file 4 — Reporting Summary [file 41467_2024_55059_MOESM4_ESM.pdf]

nature portfolio

Corresponding author(s): Kellie N. Smith, Hongkai Ji

Last updated by author(s): Jun 4, 2024

Reporting Summary

Nature Portfolio wishes to improve the reproducibility of the work that we publish. This form provides structure for consistency and transparency in reporting. For further information on Nature Portfolio policies, see our Editorial Policies and the Editorial Policy Checklist.

Statistics

For all statistical analyses, confirm that the following items are present in the figure legend, table legend, main text, or Methods section.

n/a	Confirmed	
☐	☒	The exact sample size (*n*) for each experimental group/condition, given as a discrete number and unit of measurement
☐	☒	A statement on whether measurements were taken from distinct samples or whether the same sample was measured repeatedly
☐	☒	The statistical test(s) used AND whether they are one- or two-sided<br>*Only common tests should be described solely by name; describe more complex techniques in the Methods section.*
☐	☒	A description of all covariates tested
☐	☒	A description of any assumptions or corrections, such as tests of normality and adjustment for multiple comparisons
☐	☒	A full description of the statistical parameters including central tendency (e.g. means) or other basic estimates (e.g. regression coefficient) AND variation (e.g. standard deviation) or associated estimates of uncertainty (e.g. confidence intervals)
☐	☒	For null hypothesis testing, the test statistic (e.g. *F*, *t*, *r*) with confidence intervals, effect sizes, degrees of freedom and *P* value noted<br>*Give P values as exact values whenever suitable.*
☒	☐	For Bayesian analysis, information on the choice of priors and Markov chain Monte Carlo settings
☒	☐	For hierarchical and complex designs, identification of the appropriate level for tests and full reporting of outcomes
☐	☒	Estimates of effect sizes (e.g. Cohen's *d*, Pearson's *r*), indicating how they were calculated

Our web collection on statistics for biologists contains articles on many of the points above.

Software and code

Policy information about availability of computer code

Data collection	No software was used for data collection
Data analysis	R v.4.0.2, Seurat, SAVER, scVelo, Python 3.6. Scripts to reproduce the analyses used in this study are available at https://github.com/BKI-immuno-KNS/MANAscore

For manuscripts utilizing custom algorithms or software that are central to the research but not yet described in published literature, software must be made available to editors and reviewers. We strongly encourage code deposition in a community repository (e.g. GitHub). See the Nature Portfolio guidelines for submitting code & software for further information.

Data

Policy information about availability of data

All manuscripts must include a data availability statement. This statement should provide the following information, where applicable:
- Accession codes, unique identifiers, or web links for publicly available datasets
- A description of any restrictions on data availability
- For clinical datasets or third party data, please ensure that the statement adheres to our policy

All processed and de-identified single-cell data from the neoadjuvant-treated human samples are available in the Gene Expression Omnibus with accession number GSE176022. In addition to the processed single cell data, the raw scRNA-seq–TCR-seq data are available in the European Genome-phenome Archive with accession number EGAS00001005343. Owing to the personal, sensitive and inherently identifying nature of raw genomic data, access to raw RNA-seq–TCR-seq data is

controlled and may require institutional data use agreements. Full instructions to apply for data access can be found at https://ega-archive.org/access/data-access. All data needed to evaluate the conclusions in the manuscript are present in the paper or the supplementary materials.

Research involving human participants, their data, or biological material

Policy information about studies with human participants or human data. See also policy information about sex, gender (identity/presentation), and sexual orientation and race, ethnicity and racism.

Reporting on sex and gender	No sex- and gender-based analyses have been performed
Reporting on race, ethnicity, or other socially relevant groupings	No race, ethnicity or other socially relevant groupings or analyses were performed.
Population characteristics	Caushi et al., a cohort of 15 non-small cell lung cancer (NSCLC) patients who were treated with neoadjuvant anti-PD-1, including 6 responders an 9 non-responders.<br>Oliveira et al., a cohort of 3 treatment naive melanoma patients.<br>Luoma et al., a cohort of oral cancer patients treated with neoadjuvant anti-PD-1 or anti-PD-1/CTLA-4, with 3 patients with validated tumor-reactive clones<br>Lowery et al., a cohort of metastatic cancer patients, including breast cancer, colorectal cancer and melanoma, with validated MANA-specific TIL<br>Ryu et al., a cohort of three MCPyV-postive Merkel cell carcinoma patients, with MCPyV-specific identified by CITEseq with various barcodes MCPyV peptide-HLA multimers matched to each patient's HLA genotype.
Recruitment	No recruitment was performed for this study
Ethics oversight	None of the work presented here included identifiable patient data and/or used publicly-available datasets

Note that full information on the approval of the study protocol must also be provided in the manuscript.

Field-specific reporting

Please select the one below that is the best fit for your research. If you are not sure, read the appropriate sections before making your selection.

☒ Life sciences ☐ Behavioural & social sciences ☐ Ecological, evolutionary & environmental sciences

For a reference copy of the document with all sections, see nature.com/documents/nr-reporting-summary-flat.pdf

Life sciences study design

All studies must disclose on these points even when the disclosure is negative.

Sample size	Sample size was determined based on the availability of published single cell transcriptomic data with identified tumor-reactive TIL
Data exclusions	One tumor sample from the Lowery et al dataset was excluded due to significant batch effects
Replication	Replication is not relevant to our study owing to the retrospective nature of the work (i.e. using published data)
Randomization	Randomization is not relevant to our study owing to the retrospective nature of the work (i.e. using published data)
Blinding	Blinding is not relevant to our study owing to the retrospective nature of the work (i.e. using published data)

Reporting for specific materials, systems and methods

We require information from authors about some types of materials, experimental systems and methods used in many studies. Here, indicate whether each material, system or method listed is relevant to your study. If you are not sure if a list item applies to your research, read the appropriate section before selecting a response.

Materials & experimental systems

n/a	Involved in the study
☒	☐ Antibodies
☒	☐ Eukaryotic cell lines
☒	☐ Palaeontology and archaeology
☒	☐ Animals and other organisms
☒	☐ Clinical data
☒	☐ Dual use research of concern
☒	☐ Plants

Methods

n/a	Involved in the study
☒	☐ ChIP-seq
☒	☐ Flow cytometry
☒	☐ MRI-based neuroimaging

Plants

Seed stocks

Report on the source of all seed stocks or other plant material used. If applicable, state the seed stock centre and catalogue number. If plant specimens were collected from the field, describe the collection location, date and sampling procedures.

Novel plant genotypes

Describe the methods by which all novel plant genotypes were produced. This includes those generated by transgenic approaches, gene editing, chemical/radiation-based mutagenesis and hybridization. For transgenic lines, describe the transformation method, the number of independent lines analyzed and the generation upon which experiments were performed. For gene-edited lines, describe the editor used, the endogenous sequence targeted for editing, the targeting guide RNA sequence (if applicable) and how the editor was applied.

Authentication

Describe any authentication procedures for each seed stock used or novel genotype generated. Describe any experiments used to assess the effect of a mutation and, where applicable, how potential secondary effects (e.g. second site T-DNA insertions, mosiacism, off-target gene editing) were examined.
